# Esculetin Provides Neuroprotection against Mutant Huntingtin-Induced Toxicity in Huntington’s Disease Models

**DOI:** 10.3390/ph14101044

**Published:** 2021-10-13

**Authors:** Letizia Pruccoli, Carlo Breda, Gabriella Teti, Mirella Falconi, Flaviano Giorgini, Andrea Tarozzi

**Affiliations:** 1 Department for Life Quality Studies, University of Bologna, 47921 Rimini, Italy; 2 Department of Genetics and Genome Biology, University of Leicester, Leicester LE1 7RH, UK; carlo.breda@dmu.ac.uk (C.B.); fg36@leicester.ac.uk (F.G.); 3 Leicester School of Allied Health Sciences, Faculty of Health and Life Sciences, De Montfort University, Leicester LE1 7RH, UK; 4 Department of Biomedical and Neuromotor Sciences, University of Bologna, 40126 Bologna, Italy; gabriella.teti2@unibo.it (G.T.); mirella.falconi@unibo.it (M.F.)

**Keywords:** Huntington’s disease, huntingtin, mitochondrial dysfunction, oxidative stress, neuroprotection, esculetin, *Drosophila melanogaster*

## Abstract

Huntington’s disease (HD) is a neurodegenerative disorder caused by an abnormal CAG trinucleotide repeat expansion within exon 1 of the huntingtin (HTT) gene. This mutation leads to the production of mutant HTT (mHTT) protein which triggers neuronal death through several mechanisms. Here, we investigated the neuroprotective effects of esculetin (ESC), a bioactive phenolic compound, in an inducible PC12 model and a transgenic *Drosophila melanogaster* model of HD, both of which express mHTT fragments. ESC partially inhibited the progression of mHTT aggregation and reduced neuronal death through its ability to counteract the oxidative stress and mitochondria impairment elicited by mHTT in the PC12 model. The ability of ESC to counteract neuronal death was also confirmed in the transgenic *Drosophila* model. Although ESC did not modify the lifespan of the transgenic *Drosophila*, it still seemed to have a positive impact on the HD phenotype of this model. Based on our findings, ESC may be further studied as a potential neuroprotective agent in a rodent transgenic model of HD.

## 1. Introduction

Huntington’s disease (HD) is the most common monogenic neurodegenerative disorder caused by an abnormal CAG trinucleotide repeat expansion within exon 1 of the gene encoding for the huntingtin (HTT) protein [1]. An increasing number of CAG repeats is related to progressively earlier ages of disease onset, motor dysfunction, cognitive deficits, compromised daily living capacity and brain neurodegeneration [2]. When the CAG repeats are higher than 40, the gene mutation is highly penetrant and triggers a disease process that inexorably leads to full clinical expression of HD [1].

Mutant HTT (mHTT) results in the selective dysfunction and death of striatal neurons within the central nervous system through several mechanisms, including the formation of toxic mHTT aggregates, disruption of proteostasis, axonal transport, transcription, translation and direct toxicity of mHTT fragments [3,4,5,6]. In this line, the dopamine biosynthesis pathway seems to be critically affected in HD progression. The downregulation of dopamine biosynthesis genes, including tyrosine hydroxylase (TH), leads to decreased dopamine levels in cells expressing mHTT [7]. Recent studies have also demonstrated that mitochondrial dysfunction and bioenergetic defects are involved in the late stages of HD [8]. HD mitochondria showed reduced activity in several components of oxidative phosphorylation, including complexes II, III and IV of the electron transport chain [9]. Expanded polyQ repeats have been associated with low levels of mitochondrial adenosine triphosphate (ATP) and decreased mitochondrial adenosine diphosphate uptake [10]. In addition, mHTT aggregates have been found to impair retrograde and anterograde mitochondrial trafficking along cortical neuronal axons, leading to the disruption of mitochondrial maintenance [11]. Several studies have observed abnormal mitochondrial dynamics and fission/fusion leading to the accumulation of fragmented and damaged mitochondria, increased levels of oxidative stress/reactive oxygen species (ROS) and depleted intracellular ATP [12,13].

Work in vitro and in vivo has documented the protective role of various natural products, such as phenolic compounds via multiple cellular targets, notably in the context of neurodegenerative disorders, including HD [14]. Esculetin (ESC) is a bioactive phenolic compound found in the plant-derived Chinese medicine Cortex Fraxini that has exhibited neuroprotective effects in various neurodegenerative diseases, such as transient cerebral ischaemia, Parkinson’s disease and Alzheimer’s disease. Its effects on HD, however, have not been reported [15,16,17]. In this study, we investigated the neuroprotective effects of the phenolic coumarin ESC in HD genetic models expressing mHTT fragments with glutamine repeats higher than 40 that recapitulate the pathological features of HD. We used neuronal (PC12) cell lines derived from rat pheochromocytoma that express an inducible enhanced green fluorescent protein (EGFP)-tagged HTT exon 1 fragment with 23 (HD-Q23) or 74 (HD-Q74) glutamine repeats that shape the non-mutant and mutant form of HTT, respectively [18]. In particular, these inducible HD cell models allow an evaluation of the dynamics of mHTT formation and of the appearance of neurodegenerative events, as well as the identification of the potential therapeutic window of a neuroprotective substance [19]. To corroborate the results obtained in an in vitro genetic model of HD, we also employed an HTT93Q transgenic *Drosophila* model in which the mHTT fragment with 93 glutamine repeats is expressed using the binary GAL4/UAS system [20].

## 2. Results and Discussion

### 2.1. Neurotoxicity Induced by mHTT in HD-Q74 Cells

**Figure 1:**
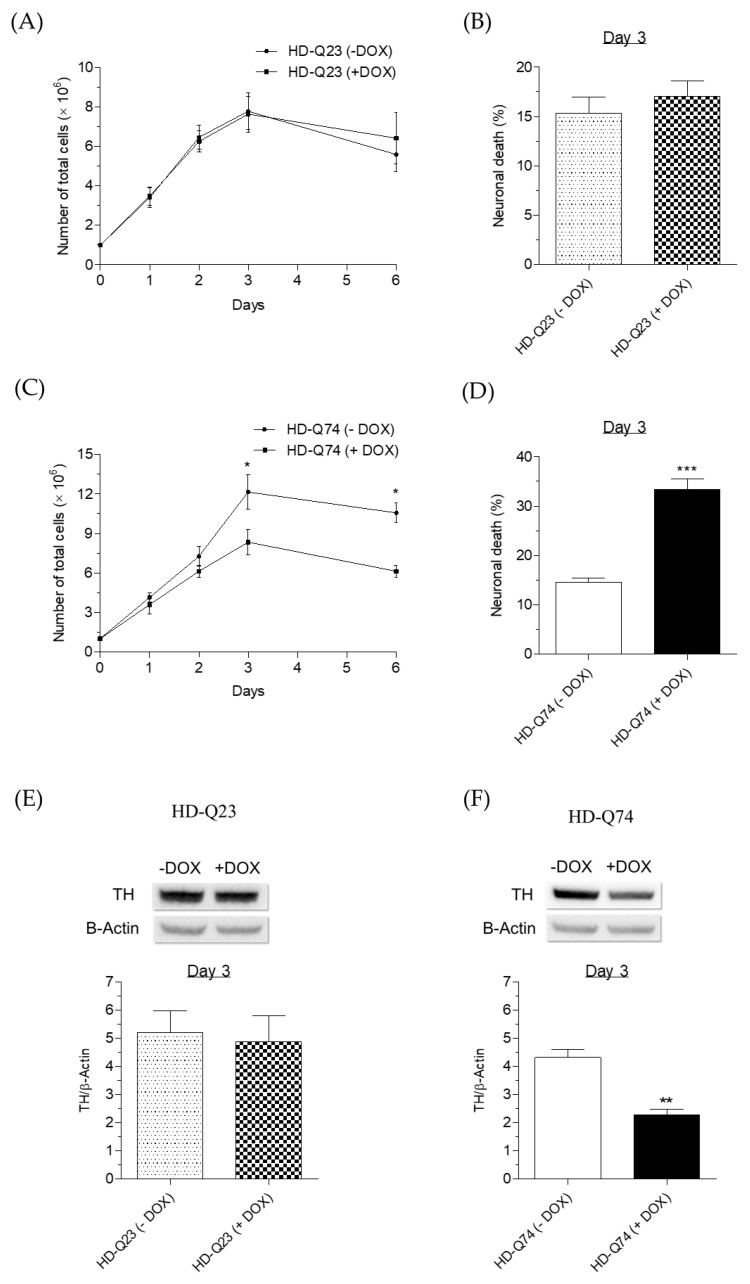
Neurotoxicity onset upon induction of mHTT in HD-Q74 cells. (**A**,**C**) HD-Q23 and HD-Q74 cells were incubated with DOX (1 µg/mL) for different times (1, 2, 3 and 6 days) and the cell proliferation was evaluated using eosin as described in the Materials and Methods. Data are expressed as numbers of total cells and reported as the mean ± SEM of at least three independent experiments (* *p* < 0.05 vs. HD-Q74 (−DOX) at one-way ANOVA with Bonferroni post hoc test); (**B**,**D**) HD-Q23 and HD-Q74 cells were incubated with DOX (1 µg/mL) for 3 days and neuronal death was evaluated using eosin as described in the Materials and Methods. Data are expressed as percentages of neuronal death and reported as the mean ± SEM of at least three independent experiments (*** *p* < 0.001 vs. HD-Q74 (−DOX) at Student’s *t*-test); (**E**,**F**) HD-Q23 and HD-Q74 cells were incubated with DOX (1 µg/mL) for 3 days and TH protein levels were evaluated by western blotting as described in the Materials and Methods. Data are expressed as TH/β-Actin and reported as the mean ± SEM of at least three independent experiments (** *p* < 0.01 vs. HD-Q74 (−DOX) at one-way ANOVA with Bonferroni post hoc test).

We initially performed a time course of neurotoxicity onset induced by mHTT to define the treatment time with ESC in HD-Q74 cells. In parallel, we adopted similar experimental conditions for HD-Q23 cells that express the non-mutant form of HTT. After an induction treatment of 3 days with DOX (1 µg/mL), we recorded significant neurotoxicity in terms of cytostatic effects (Figure 1C) and reduction of TH protein levels (Figure 1F), as well as an increase of neuronal death (Figure 1D) in HD-Q74 cells, though not in HD-Q23 cells (Figure 1A,B,E), thus confirming the ability of mHTT to decrease neuronal viability. Over time, neurotoxicity did not increase further after 6 days of induction with DOX in HD-Q74 cells. In this regard, previous studies of mHTT aggregation dynamics showed the maximum formation of fibrillar mHTT aggregates at 2–3 days post-induction by DOX in HD-Q74 cells [18,21] These experimental results suggest a causal role of fibrillar mHTT aggregates between the second and third day of induction by DOX to trigger neuronal death processes in HD-Q74 cells. However, we cannot exclude the contribution of other types of mHTT aggregates to neurotoxicity [19].

**Figure 2:**
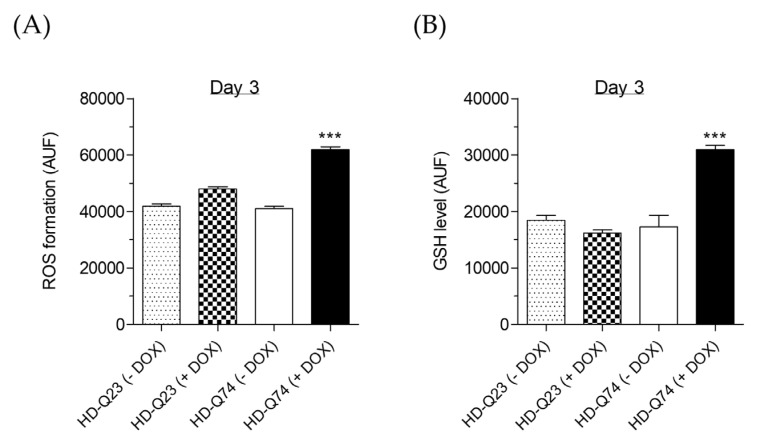
Impairment of cellular redox status upon induction of mHTT in HD-Q74 cells. The cellular redox status in terms of cytosolic ROS (**A**) and GSH (**B**) levels were evaluated in HD-Q23 and HD-Q74 cells after 3 days of incubation with DOX (1 µg/mL). At the end of incubation, ROS formation and GSH levels were evaluated using the fluorescent probes H_2_DCF-DA and MCB, respectively, as described in the Materials and Methods. Data are expressed as AUF and reported as the mean ± SEM of at least three independent experiments (*** *p* < 0.001 vs. HD-Q74 (−DOX) at one-way ANOVA with Bonferroni post hoc test).

Considering the contribution of oxidative stress evoked by mHTT to pathogenesis in mouse and human HD neurons [22], we also evaluated the redox status by measuring cytosolic ROS and glutathione (GSH) levels upon induction of mHTT. HD-Q74 cells, but not HD-Q23 cells, showed a significant increase of intracellular ROS and GSH levels after 3 days of incubation with DOX (Figure 2A,B). Interestingly, the increase of endogenous antioxidant GSH levels suggests an insufficient adaptive response to oxidative damage generated by mHTT in HD neurons. Recent combined microarray studies provide ample evidence for the induction of a protective oxidative response in HD-Q74 cells expressing mHTT [7]. Given the pathogenic impact of oxidative stress, strengthening of the endogenous antioxidant response represents a compelling therapeutic target for HD [23]. Based on this evidence, mHTT expression was induced in HD-Q74 cells with DOX for 3 days and ESC was added during the last 24 h of incubation with DOX to evaluate its protective effects against mHTT neurotoxicity. In this context, while concentrations of ESC higher than 5 µM were toxic for both HD-Q23 and HD-Q74 cells (Figure S1, Supplementary Materials), no neurotoxicity effects were observed at 5 µM, which was used in all the experiments described below.

### 2.2. Effects of ESC on mHTT Aggregation in HD-Q74 Cells

**Figure 3:**
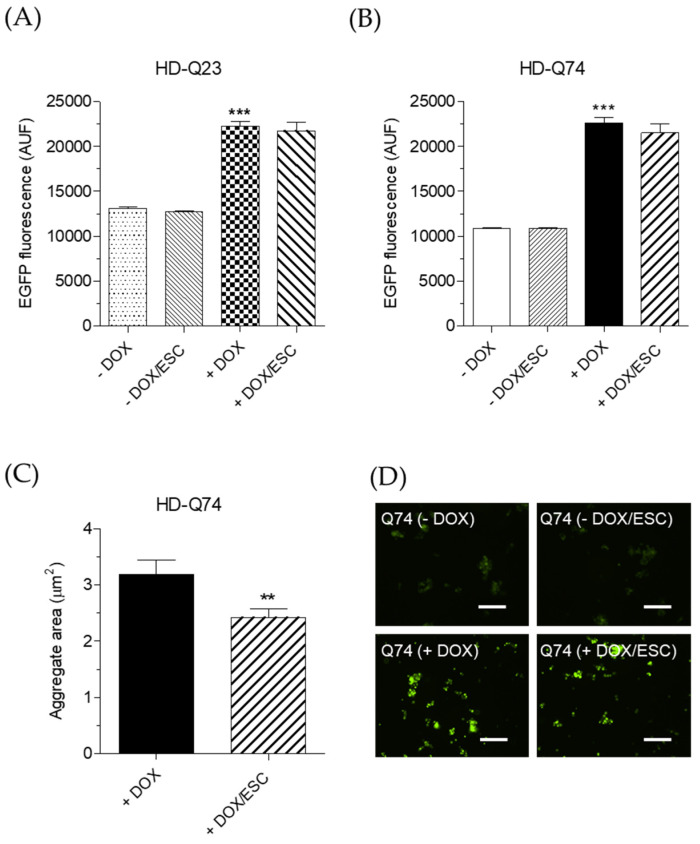
ESC inhibits mHTT aggregation in HD-Q74 cells. (**A**,**B**) HD-Q23 and HD-Q74 cells were incubated with DOX (1 µg/mL) for 3 days and ESC (5 µM) was added during the last 24 h of incubation with DOX. The fluorescence of both EGFP-tagged HTT23Q and HTT74Q aggregates was measured as described in the Materials and Methods. Data are expressed as AUF and reported as the mean ± SEM of at least three independent experiments (*** *p* < 0.001 vs. HD-Q23 (−DOX) and *** *p* < 0.001 vs. HD-Q74 (−DOX) at one-way ANOVA with Bonferroni post hoc test); (**C**) HD-Q74 cells were incubated with DOX (1 µg/mL) for 3 days and ESC (5 µM) was added during the last 24 h of incubation with DOX. The area of the fluorescent EGFP-tagged HTT74Q aggregates was measured using an inverted fluorescence microscope as described in the Materials and Methods. Data are expressed as area and reported as mean ± SEM of at least three independent experiments (** *p* < 0.01 vs. HD-Q74 (+DOX) at Student’s *t*-test); (**D**) Representative images of HTT74Q aggregates after treatment with ESC (scale bars: 100 µm).

The mHTT aggregates formation represents an important early and upstream process in the development of HD. The treatment with 5 µM of ESC during the last 24 h of incubation with DOX did not change the levels of EGFP-tagged normal HTT protein in HD-Q23 cells and EGFP- tagged mHTT protein in HD-Q74 cells (Figure 3A,B). In parallel, the same treatment recorded a significant reduction of the fluorescent aggregation area of mHTT in HD-Q74 cells (Figure 3C,D). This experimental point was not evaluated in HD-Q23 cells because the normal HTT did not aggregate according to previous studies [19]. Taken together, these results suggest the ability of ESC to partially inhibit the progression of mHTT aggregation at the neuronal level. In this regard, the results are consistent with a recent study demonstrating that ESC can potentially inhibit polyglutamine aggregation through its ability to interact with a HTT exon 1 fragment containing 36Q repeats, as determined by docking studies [24]. Among the 33 binding sites found in this HTT fragment, ESC has affinity for 12 binding sites.

### 2.3. Neuroprotective Effects of ESC against mHTT Toxicity in HD-Q74 Cells

**Figure 4:**
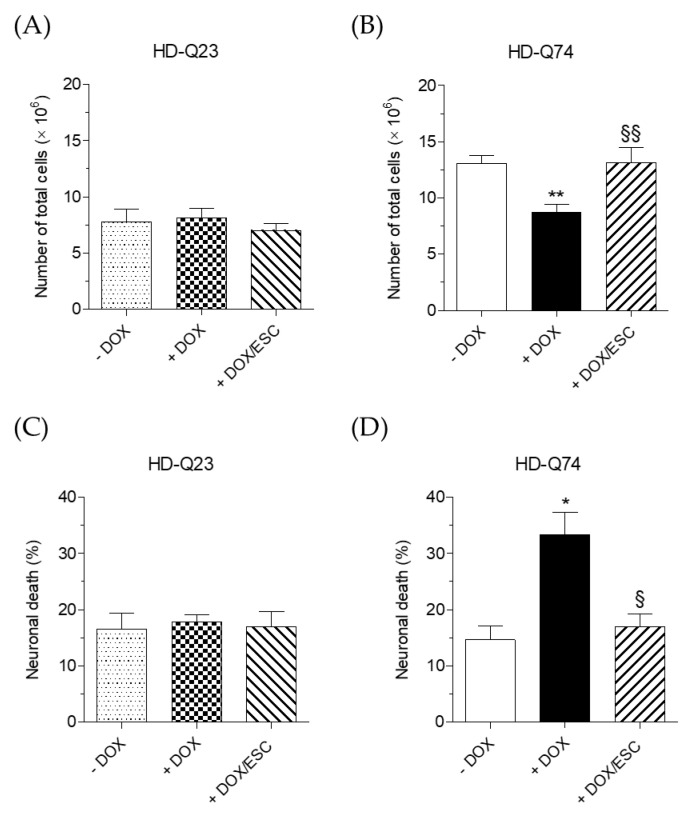
Neuroprotective effects of ESC against mHTT neurotoxicity in HD-Q74 cells. HD-Q23 and HD-Q74 cells were incubated with DOX (1 µg/mL) for 3 days and ESC (5 µM) was added during the last 24 h of incubation with DOX. Neurotoxicity, in terms of cytostatic effects (**A**,**B**) and neuronal death (**C**,**D**), was assessed using eosin as described in the Materials and Methods. Data are expressed as numbers of total cells and percentages of neuronal death and are reported as the mean ± SEM of at least three independent experiments (* *p* < 0.05 and ** *p* < 0.01 vs. HD-Q74 (−DOX), § *p* < 0.05 and §§ *p* < 0.01 vs. HD-Q74 (+DOX) at one-way ANOVA with Bonferroni post hoc test).

**Figure 5:**
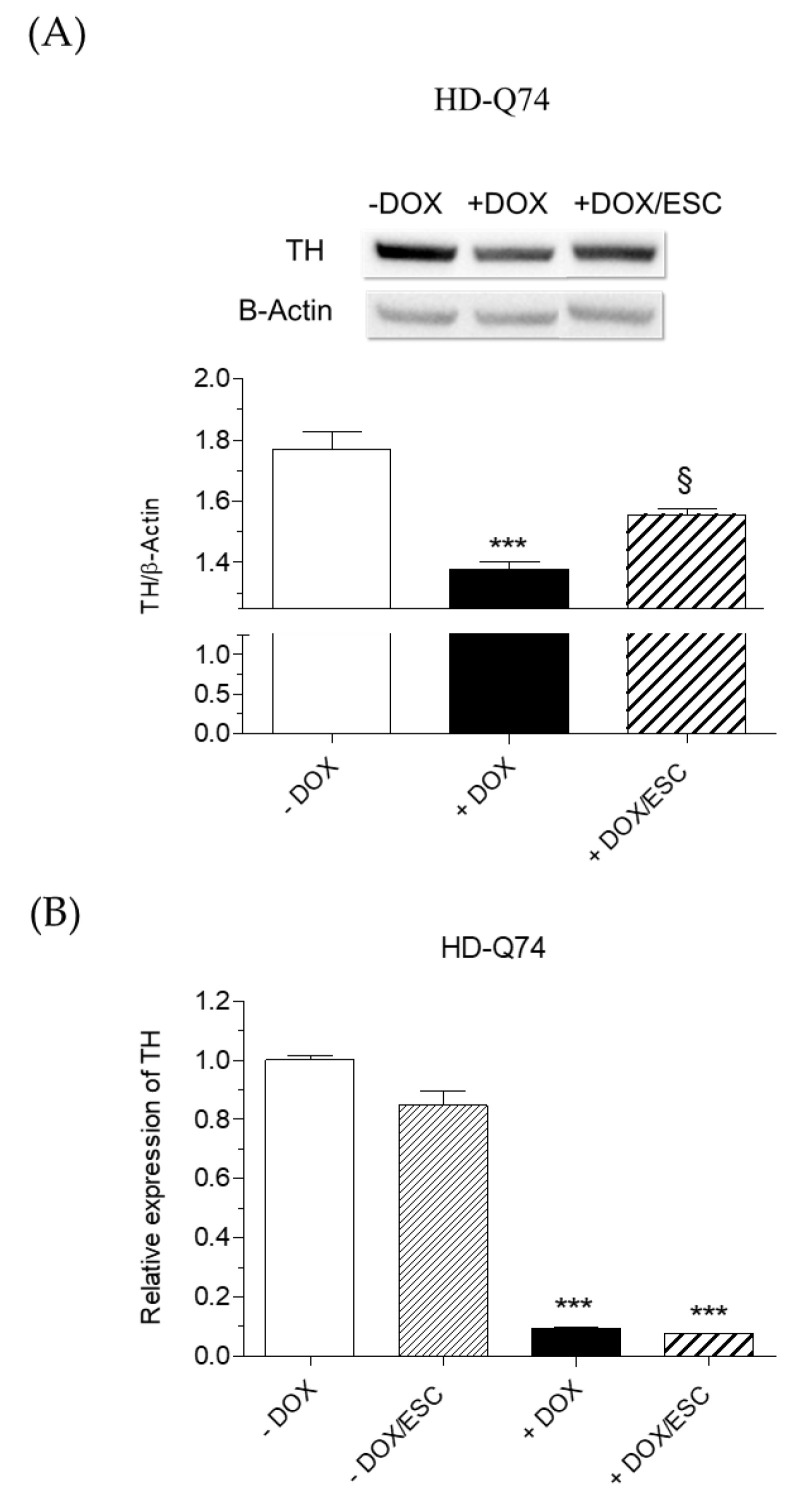
Effects of ESC on TH levels upon induction of mHTT in HD-Q74 cells. HD-Q74 cells were incubated with DOX (1 µg/mL) for 3 days and ESC (5 µM) was added during the last 24 h of incubation with DOX. TH protein levels (**A**) and gene expression (**B**) were evaluated by western blotting and RT–PCR, respectively, as described in the Materials and Methods. Data are expressed as TH protein levels and relative normalized gene expression and are reported as the mean ± SEM of at least three independent experiments (*** *p* < 0.001 vs. HD-Q74 (−DOX) and § *p* < 0.05 vs. HD-Q74 (+DOX) at one-way ANOVA with Bonferroni post hoc test).

To evaluate the neuroprotective effects of ESC against the toxicity elicited by mHTT in HD-Q74 cells, we used the same experimental conditions for evaluating its effects on mHTT aggregation. Therefore, HD-Q23 and HD-Q74 were incubated with DOX for 3 days and various concentrations of ESC (1.25, 2.5 and 5 µM) were added during the last 24 h of incubation with DOX. The treatment with 5 µM of ESC significantly counteracted the reduced cytostatic effect and the neuronal death elicited by mHTT in HD-Q74 cells (Figure 4B,D). ESC did not affect these parameters in HD-Q23 cells, confirming its neuroprotective action against mHTT neurotoxicity (Figure 4A,C). The same concentration of ESC also rescued TH protein levels (Figure 5A) but did not recover impaired *TH* gene expression (Figure 5B). These results show the ability of ESC to protect HD-Q74 cells against cell damage elicited by mHTT, which is critical for neuronal survival. In this regard, the ability of ESC to partially prevent TH impairment at the cytoplasmic level highlights its inability to protect the neurons at the transcriptional level in the nucleus. Recent studies showing the formation and aggregation of mHTT in both the nucleus and cytoplasm strengthen our hypothesis that ESC acts mainly on mHTT toxic events at the cytoplasmic level [18].

### 2.4. Effects of ESC on Mitochondrial Activity Altered by mHTT

**Figure 6:**
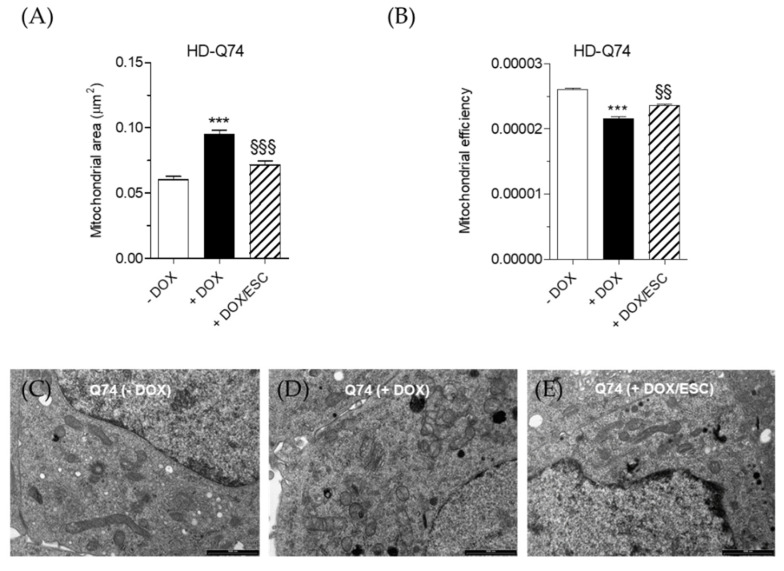
Effects of ESC on mitochondrial activity in HD-Q74 cells. Cells were incubated with DOX (1 µg/mL) for 3 days and ESC (5 µM) was added during the last 24 h of incubation with DOX. The mitochondrial area (**A**) and mitochondrial efficiency (**B**) were determined as described in the Materials and Methods. Data are reported as the mean ± SEM of at least three independent experiments (*** *p* < 0.001 vs. HD-Q74 (−DOX), §§ < 0.01 and §§§ *p* < 0.001 vs. HD-Q74 (+DOX) at one-way ANOVA with Bonferroni post hoc test). (**C**) Ultrastructural TEM image of untreated HD-Q74 cells (−DOX). Mitochondria showed a tubular shape with dense matrixes and regular cristae (scale bar: 1000 nm). (**D**) Ultrastructural TEM image of HD-Q74 cells induced with DOX (+DOX). Several mitochondria showed a round shaped morphology, extracted matrixes and swollen cristae (scale bar: 1000 nm). (**E**) Ultrastructural TEM image of HD-Q74 cells induced with DOX and treated with ESC (+DOX/ESC). Mitochondria showed a tubular shape, with dense matrixes and regular cristae (scale bar: 1000 nm).

Since the decrease of ATP levels is a main feature associated with mitochondrial dysfunction in HD [25], we evaluated both the mitochondrial area and the mitochondrial efficiency in HD-Q74 cells by assessing mitochondrial area and ATP formation/mitochondrial area ratio upon 3 days’ induction of mHTT by DOX. In particular, as shown in Figure 6A,B, the induction of mHTT caused a decline in mitochondrial efficiency despite an increase in mitochondrial area, suggesting the activation of compensation mechanisms which is known as mitohormesis [26]. Remarkably, the addition of ESC during the last 24 h of incubation with DOX significantly restored the normal mitochondrial area (Figure 6A) and rescued the mitochondrial efficiency (Figure 6B). Transmission electron microscopy (TEM) analysis allowed the evaluation of morphology at the level of each mitochondrion. High magnification images of untreated HD-Q74 cells (−DOX) showed the presence of several tubular mitochondria with dense cristae in the cytoplasm (Figure 6C). By contrast, HD-Q74 cells induced with DOX (+DOX) exhibited small and round shaped mitochondria, with a less dense matrix and swollen cristae (Figure 6D). ESC treatment rescued the round shaped morphology with tubular mitochondria characterized by the presence of dense cristae in the cytoplasm (Figure 6E). These findings therefore show the ability of ESC to protect the mitochondria from damage induced by mHTT. Several studies support these results showing the neuroprotective effects of ESC against neurodegeneration elicited by neurotoxins, such as oligomers of amyloid beta peptides and methyl-4-phenyl-1,2,3,6-tetrahydropyridine, that target mitochondria [16,27]. Based on our results, we can hypothesize some mechanisms of mitochondrial protection mediated by ESC, including its ability to prevent the formation of mHTT aggregates toxicity toward mitochondria and thereby protect their activity. In this regard, a recent study described ESC as an ATP regulator, considering its ability to protect the mitochondria against the impairment induced by 1-methyl-4-phenyl-1,2,3,6-tetrahydropyridine and rotenone via estrogen receptor-related receptors in a Parkinson’s disease mouse model [28].

### 2.5. Effects of ESC on Cellular Redox Status Altered by mHTT

**Figure 7:**
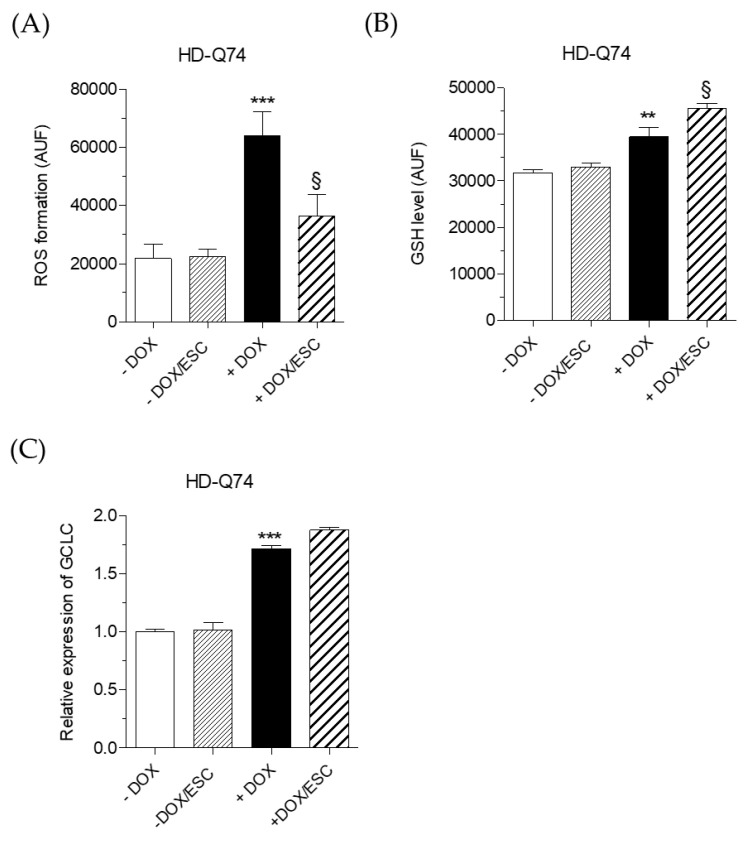
ESC improves the cellular redox status altered by mHTT in HD-Q74 cells. HD-Q74 cells were incubated with DOX (1 µg/mL) for 3 days and ESC (5 µM) was added during the last 24 h of incubation with DOX. ROS formation (**A**) and intracellular GSH levels (**B**) were evaluated using the fluorescent probes H_2_DCF-DA and MCB, respectively, as described in the Materials and Methods; (**C**) *GCLC* gene expression was evaluated by RT–PCR. Data are reported as the mean ± SEM of at least three independent experiments (** *p* < 0.01 and *** *p* < 0.001 vs. HD-Q74 (−DOX), § *p* < 0.05 vs. HD-Q74 (+DOX) at one-way ANOVA with Bonferroni post hoc test).

Oxidative stress due to accumulation of mHTT aggregates, imbalance in oxidant and antioxidant species and impairment in the electron transport chain plays a crucial role in the progression of HD [29]. The cellular redox status, in terms of ROS formation and intracellular GSH level, was evaluated in both HD-Q23 and HD-Q74 cells. The treatment of 24 h with 5 µM of ESC significantly counteracted ROS formation (Figure 7A) and enhanced GSH increase (Figure 7B), induced by the expression of mHTT. The effects of ESC on GSH levels were not associated with a potentiated expression of genes involved in GSH synthesis. As shown in Figure 7C, ESC did not modify the upregulation of the glutamate-cysteine ligase catalytic (*GCLC*) gene, which plays an important role in the first step of GSH synthesis [30], upon induction of mHTT—excluding the possibility that ESC is acting at the transcriptional level. Taken together, these results suggest an intrinsic antioxidant activity of ESC against the ROS formation generated by mHTT in HD-Q74 cells. In this regard, previous studies demonstrated that ESC is a redox active phenolic compound that directly scavenges various radical species [31,32]. Furthermore, we recently demonstrated the ability of ESC to cross the cell membrane and reach the cytoplasm of neuronal cells where it engages in its radical scavenger activity [27]. Although ESC did not change the basal levels of GSH in HD-Q74 cells in the absence of DOX, ESC doubled the increase of GSH upon induction of mHTT suggesting the contribution of indirect mechanisms to its antioxidant activity against the oxidative stress triggered by mHTT. Given that the synthesis of GSH occurs via a two-step ATP-requiring enzymatic process, it is plausible that ESC enhances GSH synthesis through its ability to restore the mitochondrial efficiency of ATP synthesis.

### 2.6. Neuroprotective Effects of ESC in HTT93Q Transgenic Drosophila

**Figure 8:**
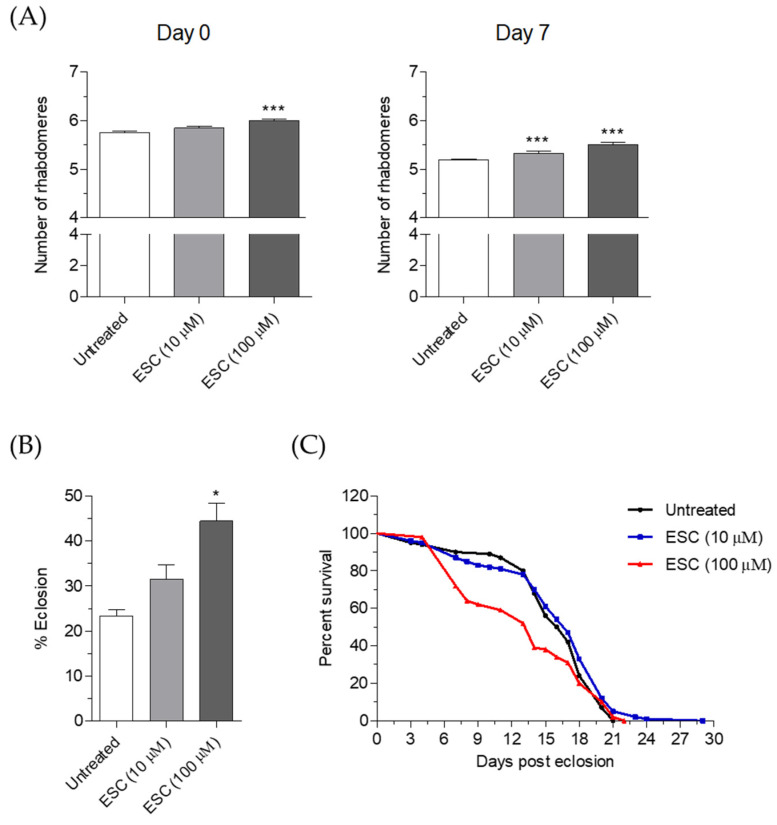
ESC ameliorates neurodegeneration in transgenic HTT93Q flies. (**A**) *Drosophila* expressing HTT93Q exon 1 pan-neuronally were fed with ESC at different concentrations during development and as adults. Rhabdomeres were scored via the pseudopupil assay at day 0 (newly emerged flies) and at day 7 (adult flies) post eclosion as described in the Materials and Methods. Data are expressed as the mean rhabdomere count per ommatidium ± SEM (*n* = 12 per condition) (*** *p* < 0.001 vs. HTT93Q flies untreated at one-way ANOVA with Newman–Keuls post hoc test). (**B**) Crosses were set up in food containing either 10 or 100 µM of ESC. The number of adult females and males emerging from the pupal case was scored using eclosion analysis as described in the materials and methods. Data are expressed as mean ± SEM (*n* = 100 per condition) (* *p* < 0.05 vs. HTT93Q flies untreated at one-way ANOVA with Newman–Keuls post hoc test). (**C**) Crosses were carried out to obtain the desired genotype and ESC was mixed in fly food at different concentrations for growing adult flies as described in the materials and methods. Data are expressed as mean ± SEM (*n* = 100 per condition).

The neuroprotective effects of ESC were further explored using fruit flies expressing a mutant HTT exon 1 fragment (HTT93Q) pan-neuronally via the *elav*GAL4 driver [20]. These flies exhibit several phenotypes that recapitulate HD symptoms, including progressive neurodegeneration (assessed by scoring loss of the photoreceptor neurons known as rhabdomeres), impaired eclosion from the pupal case and reduced lifespan. Rhabdomere number was assessed on both newly emerged flies treated with ESC during development (day 0) and aged adults treated with ESC during the first 7 days post eclosion (day 7). ESC significantly ameliorated rhabdomere degeneration in HTT93Q flies when administered either during the larval stage or in adult flies. Notably, 100 µM of ESC significantly reduced neurodegeneration when administered in food during the larval stage (Figure 8A, left panel) and ameliorated the neurodegeneration at all concentrations tested (10 and 100 µM) when administered in adult flies (Figure 8A, right panel). Furthermore, a protective effect was observed for ESC when eclosion was analysed; a greater proportion of HD flies emerged from their pupal case when treated with 100 µM of ESC compared to the untreated HD controls (Figure 8B). ESC feeding did not significantly extend shortened median lifespan in HTT93Q flies (Figure 8C). In contrast to a concentration of 10 µM, a concentration of 100 µM of ESC further shortened the lifespan of HTT93Q flies. Taken together, these results indicate that ESC ameliorates rhabdomere loss when the HTT93Q flies are supplemented at both larval and adult stages, suggesting its ability to prevent and counteract neuronal death along the different life stages. However, the neuroprotective effects were correlated with the concentrations of ESC only during supplementation in the adult stage, highlighting a strictly neuroprotective mechanism in this life stage against the toxicity mediated by mHTT fragments. These findings corroborate the neuroprotective effects of ESC recorded in HD-Q74 cells and support our hypothesis that ESC mainly protects critical components of neurons, such as mitochondria, from mHTT toxic events. With respect to adult lifespan, we did not observe positive effects of ESC in HTT93Q flies. This suggests that ESC may modulate the phenotype associated with selective neuronal death but not the lifespan in HTT93Q flies. In this regard, 100 µM of ESC showed negative effects on HTT93Q fly lifespan, which may indicate toxic effects due to a nonoptimal dose.

## 3. Materials and Methods

### 3.1. Chemicals

**Figure 9:**
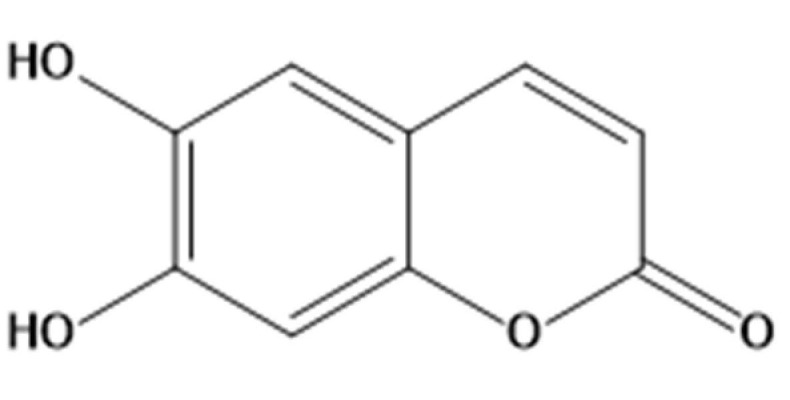
Chemical structure of ESC.

The phenolic coumarin ESC (Figure 9) was purchased from Sigma-Aldrich (Sigma-Aldrich, St. Louis, MO, USA) and resuspended in dimethyl sulfoxide. Horse serum (HS), glutamine, penicillin, streptomycin, G-418, hygromycin B, eosin B, 2′,7′-dichlorodihydrofluorescein diacetate (H_2_DCF-DA), monochlorobimane (MCB), leupeptin, PMSF, protease/phosphatase inhibitors cocktail and anti-β-Actin antibody were purchased from Sigma-Aldrich (Sigma-Aldrich). Dulbecco’s Modified Eagle’s Medium (DMEM) and fetal bovine serum (FBS) were purchased from Euroclone (Euroclone, Milan, Italy). TH antibody was purchased from Santa Cruz (Santa Cruz Biotecnology, Dallas, TX, USA). All chemicals used were of high purity analytical grade.

### 3.2. Cell Cultures

Inducible rat pheochromocytoma (PC12) cells expressing enhanced green fluorescent protein EGFP-tagged exon 1 of the HTT gene with 23 or 74 glutamine repeats (HD-Q23 or HD-Q74), driven by a doxycycline-dependent Tet-On promoter, were kindly provided by David Rubinsztein (University of Cambridge, Cambridge, UK). Cells were grown in DMEM supplemented with 10% HS, 5% FBS, 2 mM glutamine, 100 U/mL penicillin, 100 µg/mL streptomycin, 50 µg/mL G-418 and 70 µg/mL hygromycin B at 37 °C in a humidified incubator with 5% CO_2_. For the experiments, cells were incubated with DOX (1 µg/mL) for 3 days and ESC (5 µM) was added during the last 24 h of incubation with DOX for all the experiments.

### 3.3. Determination of Cell Proliferation and Neuronal Death

HD-Q23 and HD-Q74 cells were seeded in 100 mm dishes at 2 × 10^6^ cells/dish and incubated with DOX (1 µg/mL) for different lengths of time (1, 2, 3 and 6 days) at 37 °C in 5% CO_2_ to obtain the expression of the HTT exon 1 fragment. At the end of incubation, cell proliferation and neuronal death were evaluated using the dye eosin B. Briefly, cell suspensions were mixed with eosin (1:5) and then visually examined to differentiate viable and dead cells. Cells were counted to quantify proliferation and death. Data are expressed as number of total cells and percentage of neuronal death.

### 3.4. Determination of TH Protein Level

HD-Q23 and HD-Q74 cells were seeded in 100 mm dishes at 2 × 10^6^ cells/dish and incubated with DOX (1 µg/mL) for 3 days at 37 °C in 5% CO_2_ to obtain the expression of the HTT exon 1 fragment. ESC (5 µM) was added during the last 24 h of incubation with DOX. At the end of incubation, cells were pelleted and resuspended in complete lysis buffer containing leupeptin (2 µg/mL), PMSF (100 µg/mL) and a cocktail of protease/phosphatase inhibitors (100×). Small amounts were removed for the determination of the protein concentration using the Bradford method. The protein lysates (50 μg per sample) were separated by 12% SDS polyacrylamide gels (Bio-Rad Laboratories, Hercules, CA, USA) and transferred onto 0.45 μm nitrocellulose membranes, which were probed with primary TH antibody (1:1000) and secondary antibody. ECL reagents (Pierce, Rockford, IL, USA) were utilized to detect targeted bands. The same membranes were probed with anti-β-Actin (1:1000) and secondary antibody. Data were analyzed by densitometry, using Image Lab software (Bio-Rad Laboratories). Data are expressed as the ratio between TH and β-Actin protein levels.

### 3.5. Determination of mHTT Protein Aggregation

HD-Q23 and HD-Q74 cells were seeded in 100 mm dishes at 2 × 10^6^ cells/dish and incubated with DOX (1 µg/mL) for 3 days at 37 °C in 5% CO_2_ to induce expression of the HTT exon 1 fragment. ESC (5 µM) was added during the last 24 h of incubation with DOX. At the end of the incubation, cells were counted to obtain a 5 × 10^5^ cells/mL suspension from each dish. Then, 100 µL of the cell suspension were added to a black 96-well plate. Mutant HTT protein aggregation was detected (excitation at 485 nm and emission at 535) using a VICTOR X3 multilabel plate reader. Data are expressed as arbitrary units of fluorescence (AUF). Drops of the same cell suspensions were observed under an inverted fluorescence microscope (Eclipse Ti-E, Nikon, Melville, NY, USA) at λ excitation = 485 nm and λ emission = 535 nm. Four randomly selected areas with 20–30 fluorescent aggregates for each experiment point were examined and the area of each aggregate was measured by NIS-Elements Microscope Imaging Software (Nikon). Data are expressed as area (µm^2^) per aggregate.

### 3.6. Determination of ROS Formation

HD-Q23 and HD-Q74 cells were seeded in 100 mm dishes at 2 × 10^6^ cells/dish and incubated with DOX (1 µg/mL) for 3 days at 37 °C in 5% CO_2_ to obtain the expression of the HTT exon 1 fragment. ESC (5 µM) was added during the last 24 h of incubation with DOX. At the end of the incubation, cells were counted to obtain 5 × 10^5^ cells/mL suspension from each dish. Then, 100 µL of the cell suspension and 100 µL of H_2_DCF-DA (20 µg/mL) were added to a black 96-well plate. After 30 min of incubation at room temperature, ROS formation was detected (excitation at 485 nm and emission at 535 nm) using a VICTOR X3 multilabel plate reader. Data are expressed as AUF.

### 3.7. Determination of Intracellular GSH Levels

HD-Q23 and HD-Q74 cells were seeded in 100 mm dishes at 2 × 10^6^ cells/dish and incubated with DOX (1 µg/mL) for 72 h at 37 °C in 5% CO_2_ to obtain the expression of the HTT exon 1 fragment. ESC (5 µM) was added during the last 24 h of incubation with DOX. At the end of incubation, cells were counted to obtain 5 × 10^5^ cells/mL suspension from each dish. Then, 100 µL of the cell suspension and 100 µL of MCB (100 µM) were added to a black 96-well plate. After 30 min of incubation at room temperature, intracellular GSH levels were detected (excitation at 355 nm and emission at 460 nm) using a VICTOR X3 multilabel plate reader. Data are expressed as AUF.

### 3.8. Determination of TH and GCLC Gene Expression

HD-Q74 cells were seeded in 100 mm dishes at 2 × 10^6^ cells/dish and incubated with DOX (1 µg/mL) for 3 days at 37 °C in 5% CO_2_ to achieve the expression of HTT exon 1 fragment. ESC (5 µM) was added during the last 24 h of incubation with DOX. At the end of incubation, cells were centrifugated at 6000 rpm for 5 min at 4 °C to obtain a pellet and RNA was isolated using a PureLink RNA Mini Kit (Life Technologies, Carlsbad, CA, USA). RNA was quantified spectrometrically with the NanoVue Plus spectrophotometer (GE Healthcare, Chicago, IL, USA) for quantitative Real Time–PCR (qRT–PCR). Reverse transcription of RNA was performed using the SuperScript VILO MasterMix (Invitrogen, Carlsbad, CA, USA). First-strand cDNA synthesis was performed with 1 µg of total RNA. The CFX Connect Real Time System (Bio-Rad) was used for amplification and real time quantification. PCR reactions of each sample were performed in triplicate in a final volume of 20 µL in a 96-well plate. The PCR mixture containing 2 µL of cDNA (10 ng/µL), 10 µL of SYBR Select Master Mix (Invitrogen) and primers at a final concentration of 300 nM was amplified with the following conditions: initial denaturation at 95 °C for 3 min, followed by 40 amplification cycles at 95 °C for 3 s and 60 °C for 30 s. After the amplification reaction, melting curve analysis was performed starting at 65 °C and increasing to 95 °C with five acquisitions/°C. Relative normalized expression were calculated according to the 2^−ΔΔCq^ method with *β-Actin* and *Ywhaz* (Life Technologies) as reference genes and uninduced cells (−DOX) as control. The primer sequences used in this study are listed in Table 1.

### 3.9. Determination of ATP Levels

HD-Q74 cells were seeded in an opaque-walled 96-well plate at 5 × 10^3^ cells/well and incubated with DOX (1 µg/mL) for 72 h at 37 °C in 5% CO_2_ to obtain the expression of HTT exon 1 fragment. ESC (5 µM) was added during the last 24 h of incubation with DOX. At the end of incubation, 100 µL of CellTiter-Glo reagent (Promega, Madison, WI, USA) were added to each well and the contents were mixed for 2 min on an orbital shaker to induce cell lysis. Subsequently, the plate was incubated at room temperature for 10 min to stabilize the luminescent signal. The luminescent ATP levels were detected using a VICTOR X3 luminometer (PerkinElmer, Waltham, MA, USA) and converted in µM using an ATP standard curve, which was generated according to the manufacturer’s guidelines (Promega). The mitochondrial efficiency is expressed as the ratio between ATP level (µM) and mitochondrial area (µm^2^) per viable cell. The mitochondrial area was calculated as described in Section 3.10.

### 3.10. Ultrastructure Analysis by TEM

HD-Q74 cells were fixed in 2.5% glutaraldehyde in 0.1 M cacodylate buffer for 2 h at 4 °C and post fixed in 1% OsO4 in 0.1 M cacodylate buffer for 30 min at room temperature. Dehydration was completed in graded acetone–water solution and embedding in epoxy resin (Fluka, Sigma-Aldrich, St. Louis, MO, USA). Ultrathin sections (100 nm) were cut using a Diatome (Diatome, Hatfield, PA, USA) diamond knife on a Reichert-Jung ultramicrotome (Ultracut E, Reichert G, Wien, Austria) and were stained with alcoholic uranyl acetate and Reynold’s lead citrate. Sections were observed by a TEM CM10 Philips (FEI Company, Eindhoven, The Netherlands) at an accelerating voltage of 80 kV. Images were recorded with a Megaview III digital camera (FEI Company, Eindhoven, The Netherlands). Mitochondrial morphometric analysis was carried out on 100 nm ultrathin slices by ITEM software (FEI Company, Eindhoven, The Netherlands). 30 cell sections from each sample, from five to six randomly chosen regions, were acquired at 19,000×. The mitochondrial area (µm^2^) was calculated in these regions and expressed as an average value.

### 3.11. Drosophila Melanogaster

Flies were maintained in standard maize food at 25 °C in light/dark cycles of 12/12 h. The *elav*GAL4 fly stock was obtained from the Bloomington Drosophila Stock Center (USA). The transgenic lines expressing mHTT exon 1 fragment (HTT93Q) were a gift from Larry Marsh and Leslie Thompson, University of California, Irvine, USA. Crosses were set up between male flies carrying the *elav*GAL4 driver and virgin females carrying the HTT93Q transgene. In the following F1 only females expressed HTT93Q.

### 3.12. Drosophila Compound Feeding

For compound feeding experiments, maize food was heated until liquid and distributed into vials. ESC was freshly prepared in DMSO as 1000× stock and added to the food at a final concentration of 10 and 100 µM.

#### 3.12.1. Pseudopupil Analysis

This assay allows rapid visualization of rhabdomere arrangement in the ommatidia of the compound eye which is a direct measurement of the number of surviving photoreceptor neurons. Newly emerged HTT93Q exon 1 flies were transferred to vials containing DMSO or ESC treated food, which was changed daily for 7 days. At day 7, flies were anaesthetized with CO_2_, their heads removed and mounted face-up on microscope slides. A Nikon Optiphot-2 microscope at 50× magnification was used for counting rhabdomeres from approximately 100 ommatidia per fly and 12 flies per treatment. The pseudopupil assay was also performed on newly emerged adults at day 0. Crosses were set up directly into vials containing DMSO or ESC treated food. On the day of emergence from the pupal case, the rhabdomeres of HTT93Q exon 1 flies were quantified as described above.

#### 3.12.2. Eclosion Analysis

Five males carrying the sex-linked *elav*GAL4 driver were crossed to five virgin females homozygous for the UAS-HTT93Q transgene generating experimental females and control males in the F1 generation. Flies were allowed to lay eggs on vials containing DMSO or ESC treated food and parental flies were removed after 5 days. The number of adult females and males emerging from the pupal case in each vial was counted every day for 10 days. Eclosion percent was determined by the following calculation: (number of female flies/number of male flies) × 100.

#### 3.12.3. Longevity Analysis

Virgin HTT93Q exon 1 females were collected and kept in groups of 10 in separate vials containing DMSO or ESC treated food. Vials were inspected and changed every day. The number of flies remaining alive was scored. Data are expressed as percent survival.

### 3.13. Statistical Analysis

Data are reported as the mean ± standard error mean (SEM) of at least three independent experiments. Statistical analysis was performed using Student’s *t*-test, one-way ANOVA with Bonferroni or Newman-Keuls post hoc test. Differences were considered significant at *p* < 0.05. Analysis was performed using PRISM 5 software (GraphPad Software, La Jolla, CA, USA).

## 4. Conclusions

ESC exerts neuroprotective effects against the toxicity induced by mHTT in HD-Q74 cells. In particular, ESC restored mitochondria impairment and reduced neuronal death through its ability to counteract ROS formation elicited by mHTT. The ability of ESC to counteract neuronal death was confirmed in HTT93Q flies, an adult fly model of HD. Although ESC did not modify the adult fly lifespan, it still seemed to have a positive impact on the phenotype of HTT93Q flies at both larval and adult stages.

The findings recorded in both HD genetic models therefore suggest the ability of ESC to counteract neuronal damage elicited by mHTT fragments with 74 or 93 glutamine repeats that closely resembles the neuropathology of HD. However, these findings have to be recapitulated in a rodent transgenic model to overcome the limitations of cell and fly models with respect to the translation of the conclusions to the human case, and to establish the ability of ESC to modify the phenotype of HD.

## Figures and Tables

**Table 1:** Primer sequences for quantitative RT–PCR.

Gene Name	Forward/Reverse	5′ to 3′ Sequence
Tyrosine Hydroxylase	For	GGAACGGTACTGTGGCTACC
	Rev	TTCAAGAAGCGGGACACG
Glutamate–Cysteine Ligase Catalytic	For	AAGCCTCCTCCTCCAAACTC
	Rev	TACCTCCATTGGTCGGAACT
Β-Actin	For	CTGGCTCCTAGCACCATGA
	Rev	TAGAGCCACCAATCCACACA
Ywhaz	For	AAATGAGCTGGTGCAGAAGG
	Rev	GGCTGCCATGTCATCGTAT

## Data Availability

Data is contained within the article and supplementary materials.

## Supplementary Materials

The following are available online at https://www.mdpi.com/article/10.3390/ph14101044/s1: Figure S1: Neurotoxicity of ESC in HD-Q23 and HD-Q74 cells.
