# Supplementary figures and images for: Esculetin Provides Neuroprotection against Mutant Huntingtin-Induced Toxicity in Huntington’s Disease Models
Source: Pharmaceuticals (Basel). 2021 Oct 13;14(10):1044. doi: 10.3390/ph14101044 (PMC8541026; doi:10.3390/ph14101044)

Supplementary Materials

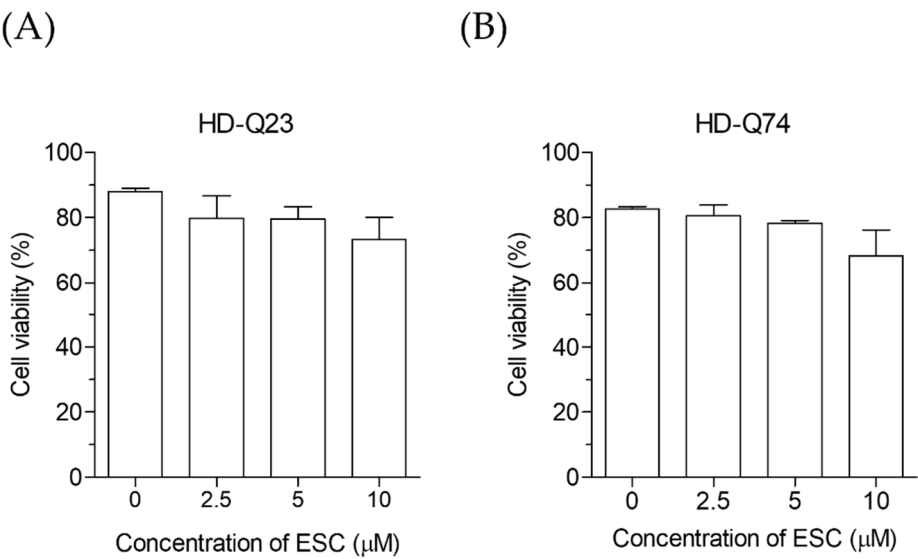

Figure S1. Neurotoxicity of ESC in HD-Q23 and HD-Q74 cells.

Supplement: Supplementary file 1 [file pharmaceuticals-14-01044-s001.zip › pharmaceuticals-1387667-supplementary.pdf]
